# Territorial status is explained by covariation between boldness, exploration, and thermal preference in a colour polymorphic lizard

**DOI:** 10.1002/ece3.70321

**Published:** 2024-09-30

**Authors:** Tyler M. Goerge, Donald B. Miles

**Affiliations:** ^1^ Department of Biological Sciences Ohio University Athens Ohio USA

**Keywords:** boldness behaviour, colour polymorphism, exploration, lizard, thermal preference

## Abstract

Colour polymorphic species often exhibit variation in morphology, physiology, and behaviour among morphs. In particular, dominance status may be signalled by the interaction between behaviour and colour morph. Behavioural traits associated with dominance include boldness, exploration, and aggression, which influence access to preferred habitat, territorial defence, and mate acquisition. In ectotherms, the social structure associated with morphs may result in the exploitation of structural niches differing in thermal quality. Hence, social interactions among morphs may generate concordant variation in thermal preference and environmental temperature. However, few studies have assessed thermal preference variation in colour polymorphic species and its covariation with behaviour. Doing so can provide insight into niche specialization and the maintenance of colour polymorphism in populations. Here, we investigated the patterns of covariation in boldness behaviour, exploratory behaviour, and thermal preference in the tree lizard, *Urosaurus ornatus*. We assessed trait variation between territorial and non‐territorial male morphs and between orange and yellow female morphs. Boldness and exploratory behaviour were repeatable in male *U. ornatus* and bolder individuals were significantly more likely to incur tail loss, a potential consequence of bold behaviour. Territorial male morphs were significantly bolder and more exploratory and preferred higher body temperatures with a narrower *T*_set_ than non‐territorial morphs. Female morphs did not vary in behavioural or thermal traits. This study highlights behavioural mechanisms that underly ecological niche segregation and variable habitat use between morphs in a colour polymorphic species.

## Graphical abstract

Male Urosaurus ornatus morphs differ in behavioural and thermal traits. Territorial males are bolder, more exploratory, and prefer higher body temperatures compared to males that do not hold territory.

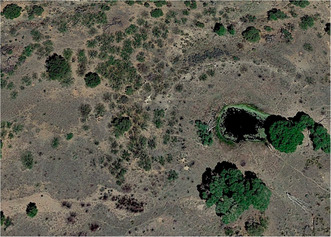

## 1 INTRODUCTION

### 1.1 Colour polymorphism and population dynamics

Colour polymorphism, or the presence of at least two distinct, genetically determined colour morphs within an interbreeding population (Huxley, 1955), has been documented in a wide range of taxa including insects (Tanaka, 2001; Willink et al., 2020), fish (Hurtado‐Gonzales et al., 2014; Maan et al., 2008), amphibians (Harkey & Semlitsch, 1988), and reptiles (Olsson et al., 2013; Roulin, 2004; Sinervo & Lively, 1996). Colour morphs often exhibit differences in morphology, physiology, whole‐organismal performance, and behaviour that may be tied to ecological factors (Brock et al., 2020; Huyghe et al., 2007; Korzan & Fernald, 2007; Kusche et al., 2015; Miles et al., 2007; Moreno, 1989). Colour polymorphic species offer valuable insights into evolutionary and ecological processes. In particular, the examination of morphs allows one to study the maintenance of phenotypic variation within a population. Within populations, disruptive selection and negative frequency‐dependent selection—both via predation (apostatic selection) and through alternative mating strategies—may favour the ability of morphs to exploit alternative habitats or resources and contribute to colour polymorphism maintenance (Bond, 2007; Gray & McKinnon, 2007; Olendorf et al., 2006; Takahashi et al., 2010). The contributions of these mechanisms to polymorphism maintenance vary among populations and species (reviewed in Gray & McKinnon, 2007; Stuart‐Fox et al., 2021); thus, quantifying trait variation among colour morphs remains imperative in improving our understanding of the situational processes that facilitate phenotypic variation and maintenance.

Several taxa have demonstrated negative frequency‐dependent selection generated by alternative reproductive strategies among morphs (Olendorf et al., 2006; Sánchez‐Guillén et al., 2017; Sinervo & Lively, 1996). Male colour morphs utilizing alternative reproductive strategies are perhaps best illustrated in the side‐blotched lizard (*Uta stansburiana*), which has three distinct throat colour morphs whose frequencies oscillate through time. Males with orange throats are hyper‐aggressive and defend large territories, males with blue throats defend smaller territories, are less aggressive, and cooperate to exclude orange males entering their territories, and males with yellow throats are not territorial and utilize “sneaker” mating tactics. These alternative reproductive strategies are maintained through negative frequency‐dependent selection yielding a rock‐paper‐scissors dynamic: blue males perform well against orange males but are more susceptible to yellow male sneaker tactics than orange males (Sinervo et al., 2007; Sinervo & Lively, 1996). In systems with multiple reproductive strategies, substantial behavioural and performance variation tends to exist among morphs (Brock & Madden, 2022; Dijkstra et al., 2008; Huyghe et al., 2009; Sinervo & Lively, 1996). Variation in phenotypes that influence predation and access to food, mating opportunities, and territory, such as locomotor performance, fighting ability, and immune response, can confer survival and reproductive advantages to particular morphs depending on environmental or situational context (Colodonato et al., 2020; Sinervo et al., 2000).

### 1.2 Behavioural and thermal variation among morphs

Behavioural variation in traits such as boldness, exploration, and aggression are thought to be major contributors to variation in dominance observed among colour morphs that exhibit alternative reproductive strategies (Kingston et al., 2003). Males of the Aegean wall lizard (*Podarcis erhardii*) have three colour morphs, which exhibit consistent boldness variation (defined as willingness to take risks). Orange morphs are the least bold, as measured by predator escape and avoidance behaviour. Orange morphs are also the least aggressive morph and perform the worst in staged contests for space (Brock et al., 2022; Brock & Madden, 2022). Boldness behaviour can influence habitat use, predation, and reproduction; for example, in yellow‐spotted monitor lizards (*Varanus panoptes*), bolder lizards have larger home ranges and spend increased time in areas with more predators than shyer lizards, while also having higher mating success (Ward‐Fear et al., 2018). Bolder individuals also tend to exhibit higher locomotor capacity (Chen et al., 2019; Goulet et al., 2017; Le Galliard et al., 2012), which can increase survivorship (Miles, 2004). However, bolder individuals also often suffer higher rates of predation (Carter et al., 2010). Another consequence of boldness variation in lizards is tail autotomy. Many lizard species can drop their tails to escape predators or aggressive conspecifics, increasing short‐term survivorship. Investigating tail loss in lizards in concordance with boldness is of interest not only because tails can serve as status‐signalling badges in lizards (Fox et al., 1990), but also because bolder individuals may have higher rates of tail loss than shyer individuals due to higher engagement in risky situations (Carter et al., 2010; Talavera et al., 2021). In addition to boldness behaviour, variation in exploration and activity rates can also influence survivorship; in Iberian wall lizards (*Podarcis hispanicus*), more exploratory lizards habituate faster to predators than do less exploratory individuals (Rodríguiz‐Prieto et al., 2011). Prior studies have demonstrated links between behaviour and fitness, though data on differing behavioural strategies among sympatric colour morphs remains scarce outside of a few model systems (but see Brock & Madden, 2022; Sreelatha et al., 2021; Yewers et al., 2016).

In ectotherms, body temperature influences biochemical processes, whole‐organism performance, and life‐history traits (Huey, 1982). Because of the thermal sensitivities of these traits, ectotherms are expected to use behavioural thermoregulation to maintain body temperatures within a range that optimizes performance (Huey & Bennett, 1987). In polymorphic species that have multiple behavioural and reproductive strategies, morphs often occupy different environmental niches (Forsman et al., 2008; i de Lanuza & Carretero, 2018; Lattanzio & Miles, 2014, 2016). When morphs utilize different microhabitats that are characterized by thermal heterogeneity, concordant variation in thermal preference may be observed (Thompson et al., 2023). However, though thermal preference has been shown to covary with lizard behaviour (Goulet et al., 2016, 2017), few studies have evaluated thermal preference variation between distinct colour morphs (but see Paranjpe et al., 2013; Thompson et al., 2023). Assessing thermal preference variation in colour polymorphic species, as well as covariation with behaviour, offers the potential to provide valuable insight into niche specialization and the maintenance of colour polymorphism.

### 1.3 Study system

**FIGURE 1:**
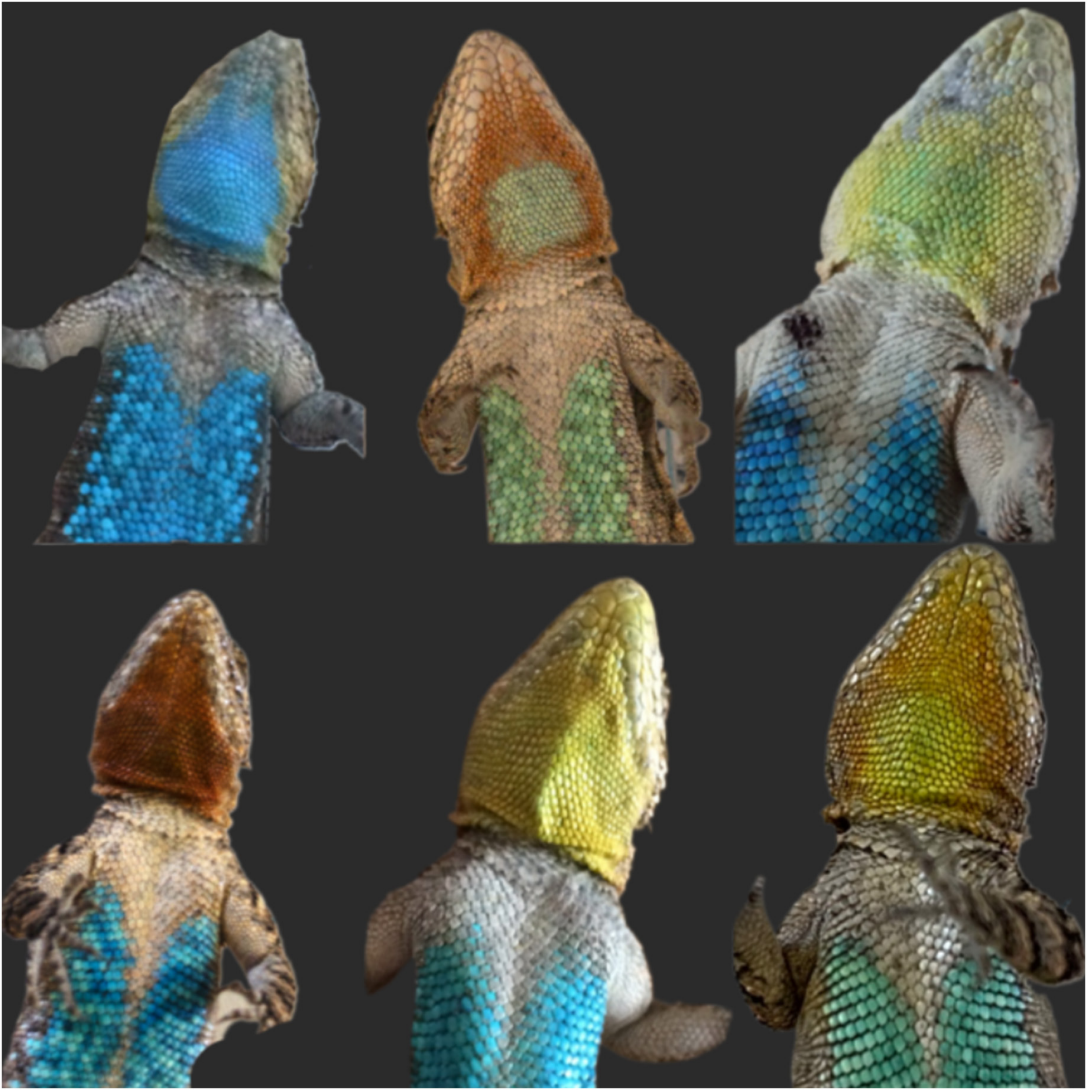
The six male morphs present at the study site. The top row (blue, orange/blue, yellow/blue) are territorial morphs and the bottom row (orange, yellow, orange/yellow) are non‐territorial morphs.

The tree lizard (*Urosaurus ornatus*) is a small‐bodied tree and rock dweller with a geographic distribution that spans the southwestern United States and northern Mexico. Male tree lizards are characterized by a throat colour polymorphism that correlates with alternative reproductive strategies. Blue males are dominant territory holders. Yellow males are satellites, defined by occupying home ranges on the periphery of dominant male territory. Orange males are sneakers, exhibiting nomadic behaviour and often behaving like females to gain close proximity to potential mates. Neither yellow nor orange males defend territories (Lattanzio & Miles, 2014, 2016; Taylor & Lattanzio, 2016). In addition, many populations contain mosaic morphs consisting of blue cores surrounded by either yellow or orange as well as yellow cores surrounded by orange (Figure 1). Despite geographic variation in which morphs are present (Goerge & Miles, 2024; Hews et al., 1997; M'Closkey et al., 1990; Thompson & Moore, 1991a), certain behavioural trends remain consistent across populations. As in blue males, yellow/blue and orange/blue males defend territory and are dominant over and more aggressive than their yellow, orange, and orange/yellow counterparts (Hews & Moore, 1995; Hover, 1985; Knapp et al., 2003; Moore & Thompson, 1990; Taylor & Lattanzio, 2016; Thompson & Moore, 1991b). Further, male *U. ornatus* morphs exhibit trophic niche and habitat use divergence. The territorial blue, yellow/blue, and orange/blue males are trophic specialists and use higher‐quality habitat—characterized by higher habitat heterogeneity, more canopy cover, and more prey items—compared to the trophic generalist yellow and orange morphs, particularly when resources are limited (Goerge & Miles, 2023; Lattanzio & Miles, 2014, 2016). Females are also polymorphic and have orange, yellow, or white throats. Female *U. ornatus* morphs differ in mate preference where orange females prefer dominant males and yellow females may avoid dominant males (Lattanzio et al., 2014). Female morphs also vary in reproductive traits such as clutch size (Zucker & Boecklen, 1990); however, it is unknown if they exhibit alternative behavioural strategies.

The goal of this study was to investigate mechanisms that may contribute to territoriality and habitat use divergence between *U. ornatus* morphs. Specifically, we assessed the covariation between behaviour and thermal preference and for trait variation between territorial (blue, yellow/blue, orange/blue) and non‐territorial (yellow, orange, orange/yellow) *U. ornatus* male morphs. We investigated boldness behaviour, defined as willingness to take risks, and exploratory behaviour, defined as activity in a novel environment. These behaviours have been shown to correlate with dominance status (Sundström et al., 2004) but are studied less than aggression and dominance. Because boldness and exploration have been demonstrated to correlate with territory size and defence (Ward‐Fear et al., 2018), we predicted that territorial males would be bolder and more exploratory than satellite and sneaker males. We also investigated tail autotomy in *U. ornatus* to determine if bolder individuals experienced higher rates of tail loss. Because territorial *U. ornatus* males occupy higher‐quality habitat with increased thermal heterogeneity compared to subordinate males (Lattanzio & Miles, 2014), we expected that territorial, blue morphs would have a higher thermal preference than subordinate/sneaker male morphs. We also investigated trait covariation among female morphs. We expected female morphs would differ in their thermal preference based on previous work on the related species *U. stansburiana* (Paranjpe et al., 2013) and the pattern of male mate preference. In particular, because orange females prefer dominant males, we predicted orange females to have higher values for thermal preference. However, social dynamics and behaviour are understudied in female *U. ornatus* compared to males, and therefore our predictions were tentative.

## 2 MATERIALS AND METHODS

### 2.1 Study site and lizard capture

**FIGURE 2:**
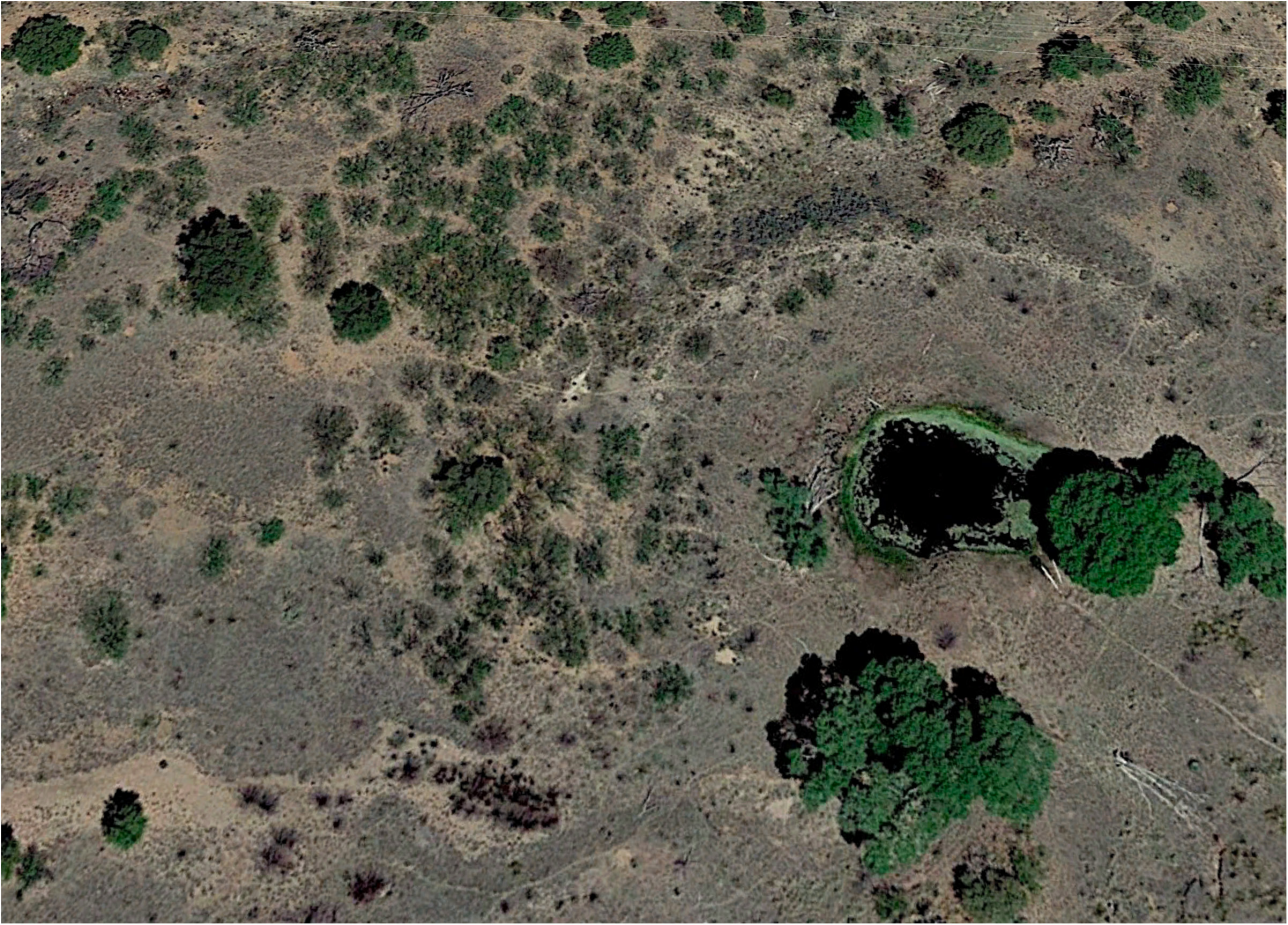
The study site at the Appleton‐Whittell Research Ranch in southeastern Arizona. Lizards were searched for on live oak and mesquite trees as well as dead tree snags.

Adult lizards were observed and captured during 6 May–31 June 2019 from a population at the Appleton‐Whittell Research Ranch of the National Audubon Society in southeastern Arizona. (31.365  N, −110.303  W; Figure 2). The population is located in a semi‐arid oak grassland. Ddult lizards are arboreal, spending the majority of their time on live oak (*Quercus* sp.), mesquite (*Prosopsis* sp.), and dead tree snags. We searched for lizards during mornings from 07:00 to 12:00. By 12:00, rising air and substrate temperatures at the study site resulted in a reduction in lizard activity (personal observation). We captured lizards using a pole and lasso. Live trees were considered high quality because they offer increased structural heterogeneity and the canopy cover provides shade compared to the open structure of snags (Lattanzio & Miles, 2014). Lizard collection was approved by the Arizona Game and Fish Department (AZ collection permit #SP622193) and the study was approved by IACUC protocol 13‐L‐021.

### 2.2 Morphological measurements and husbandry

We transported lizards to a laboratory at the Appleton‐Whittell Research Ranch. We measured snout‐vent length (SVL) using calipers (to the nearest mm) and body mass (g) using a digital Pesola® scale. We included these traits because body size may influence behaviour in lizards (de Barros et al., 2010). We also recorded the presence of regenerating tails (i.e., tail autotomy) to compare with boldness behaviour. Bolder individuals engage in risky behaviour and therefore may be more likely to have lost their tails through agonistic interactions or predator evasion (Carter et al., 2010; Talavera et al., 2021). We recorded whether the individual was missing a tail or if the individual had re‐grown the tail, evidenced by uniform grey colouration of regrown tissue. We determined the sex for each individual based on the presence of enlarged post‐anal scales in males and recorded males as territorial (blue, yellow/blue, orange/blue individuals) or non‐territorial (orange, yellow, orange/yellow individuals; Hews & Moore, 1995; Knapp et al., 2003; Lattanzio & Miles, 2014, 2016; Moore & Thompson, 1990; Taylor & Lattanzio, 2016; Thompson & Moore, 1991b). Females in the population have yellow, orange, or white throats. During captivity, lizards were housed in individual terraria and provided a thermal gradient with an upper limit at their field active body temperature (36°C) to allow for thermoregulation. We maintained lizards on a 13 h/11 h light/dark cycle to mimic local photoperiod. Nocturnal temperatures were ambient. Lizards were offered one mealworm daily and provided water ad libitum. Lizards were allowed 24 h to acclimate to laboratory conditions before we initiated the behavioural testing. Individuals were fasted for 24 h between trials.

### 2.3 Behavioural measurements

Behaviour trials were recorded on a camera (Canon PowerShot SX30 IS) to reduce observer disturbance during the experiments. Boldness was measured as the response of a lizard to a simulated predation attack, a common measure of an individual lizard's willingness to take risks (Carazo et al., 2014; López et al., 2005; Rodríguiz‐Prieto et al., 2011). We placed individuals in a 60 cm L × 30 cm W × 25 cm H enclosure with a sandy substrate and a branch for a perch on one end. The perch had a lamp suspended above to provide a basking site (heated to 36°C). Lizards were given 15 min to initiate basking on the perch, after which we chased them to the other end of the enclosure into a refuge box, simulating an act of predation. The refuge box covered cool sand and had a small opening on each side. We considered this assay to be appropriate considering that *U. ornatus* often flees predators by hiding under peeling tree bark (personal observation). We recorded lizards for 30 min after being chased into the refuge box and calculated two elements of boldness: the amount of time lizards spent perching on the branch and the amount of time they spent basking in the general heated area, but not necessarily on the branch. Bolder individuals were expected to spend more time basking and perching after the simulated predation attack than shyer individuals (Carazo et al., 2014; López et al., 2005). We replaced the sandy substrate at the end of each trial to remove potential chemical cues. The exploratory behaviour of each lizard was assessed in a novel environment: a 60 cm L × 30 cm W × 25 cm H enclosure made from insulation material. The enclosure had a plywood base upon which a grid of 18 separate 10 × 10 cm squares was drawn. The enclosure was maintained at about 36°C to match the field active body temperature of *U. ornatus*. We placed a lizard in the centre of the grid under a translucent container for 5 min, then removed the container and gave the lizard 30 min to explore the novel enclosure. We defined exploratory behaviour of an individual as the number of square transitions made in the grid over the course of the 30‐min trial. We wiped the grid with isopropyl alcohol after each trial to eliminate chemical cues.

### 2.4 Thermal preference

In a thermal gradient in a laboratory setting, lizards are able to attain a body temperature in the absence of ecological costs (predator avoidance, competition with conspecifics, etc.). Thermal preference (*T*_pref_) is expected to reflect available thermal niches to individuals in the field. We constructed a 120 cm L × 16 cm W × 20 cm H photothermal gradient with aluminium flashing affixed to a plywood base to assess individual lizards' thermal preferences. The thermal gradient had a sandy substrate base. The temperature spanned 27–45°C with temperatures maintained by 60 and 100 W heating bulbs suspended above the gradient. We placed lizards in the centre of the gradient and allowed them a 10‐min acclimation period, after which we commenced measuring body temperature (*T*_b_) with an infrared digital thermometer. We took measurements every 10 min for 60 min total. For each individual, we calculated *T*_pref_ (the mean *T*_b_ selected over the 60‐min trial) and the coefficient of variation of *T*_pref_ (*T*_pref_CV) to quantify the variability of individuals' *T*_pref_. We used *T*_pref_CV as our estimate of variability because it describes variation around a mean and our estimates of *T*_pref_ were averaged values. We also quantified a shuttling variable, calculated as the summed absolute values of the change in *T*_b_ at each 10‐min interval. Information on shuttling behaviour was included because it describes the behaviour of individuals in the thermal gradient. Individuals with low shuttling values selected and basked at a single location for the duration of the trial, whereas individuals with high shuttling values moved frequently between extreme temperature values to attain a given *T*_pref_. Thermal preference and behavioural trials were separated by 24 h to reduce stress on study individuals. After the completion of the trials, we gave lizards a unique toe clip for future identification and released them back to their site of capture. We clipped no more than one toe per foot and a maximum of three toes per individual to minimize our influence on locomotor performance (Borges‐Landáez & Shine, 2003). Toe clipping has also been shown to have minimal stress impacts on lizards (Langkilde & Shine, 2006).

### 2.5 Statistical analysis

All statistical analyses were conducted using R version 3.5.2 (R Core Team, 2019). We assessed the repeatability of exploratory behaviour (number of transitions), boldness behaviour (both time spent basking and perching), and thermal preference traits using the intraclass correlation coefficient, which measures reliability of trait scores over time. We used type ICC3 for single fixed raters using the “ICC” function in the package “psych” (Revelle, 2020). We used this model because it treats raters of repeatability—in our case, scores from behavioural and thermal trials—as fixed effects rather than randomly chosen judges of performance. Repeatability analyses entailed measuring behaviour and thermal preference on individuals twice: quantifying the suite of traits soon after their first capture, releasing lizards at the capture site following the first measurements, and waiting at least one week before recapturing individuals to conduct a second set of measurements. A one‐week period before retesting was selected to avoid potential confounding effects arising from acclimation to the laboratory. We assessed trait repeatability in males as a recapture of females would have occurred during the initiation period of egg‐laying in females and we wished to avoid interrupting reproduction.

We investigated if male status groups and female morphs differed in behavioural or thermal preference with MANCOVAs. We used Pillai's trace as a test statistic because it accommodates unequal group sizes and used Type III sum of squares (“manova” in the stats package). We included exploration (number of transitions), boldness (time spent perching and time spent basking), *T*_pref_, *T*_pref_CV, and shuttling as dependent variables. We included SVL as a covariate and excluded body mass due to its high correlation with SVL (*r* = .8). We excluded two white females from the analysis because we had fewer observations than dependent variables. We used univariate one‐way ANOVAs for post‐hoc analyses to determine which variables contributed to the separation of groups in the MANCOVA. We used Kruskal‐Wallis tests for variables with high heteroscedasticity and non‐normal residuals (basking and perching). For all traits, we applied a false discovery rate *p*‐value adjustment for multiple comparisons (Pike, 2011). We also calculated Cohen's d for each significant term to determine the effect size between status groups. We used pooled standard deviation between groups and used the function “cohens_d” in the “rstatix” package (Kassambara, 2020).

We used the function “princomp” in the stats package to conduct a principal component analysis (PCA) for males and females to describe the patterns of covariation among the thermal traits (*T*_pref_, *T*_pref_CV, shuttling) and behavioural traits (exploration, perching, basking) in relation to male status and female morph. We also included SVL and mass. The PCAs were calculated using a correlation matrix because the traits had different scales. Two principal components (PCs) were retained for males and four PCs were retained for females based on the broken‐stick criterion (Jackson, 1993). To determine if the set of behavioural and thermal traits differed between status groups or colour morphs, we compared centroid space using marginal PERMANOVAs via the function “adonis2” in the package “vegan” (Oksanen et al., 2020). We assessed which axes contributed to significant differences in centroid space using ANOVAs with the retained PCs as response variables.

We assessed if bolder lizards had higher rates of tail loss than shyer individuals (Carter et al., 2010; Talavera et al., 2021) to investigate ecological ramifications of behavioural variation and to use field data to verify results of laboratory‐based protocol. Based on boldness variation results, we used males for this analysis. We used a GPS map of our study site and captured locations to match all males that completed boldness behaviour tests and had tail breaks with their nearest neighbour that completed boldness tests but did not have a tail break. We compared males in this way to minimize confounding spatial factors that could influence tail loss, such as predator presence or habitat composition (Carter et al., 2010). We then recorded which of the two males basked longer in his boldness test and used a chi‐square test to determine if bolder males lost their tails at a higher rate than shyer males. We also used a chi‐square test to determine if territorial and non‐territorial males experienced tail breaks at different rates.

## 3 RESULTS

### 3.1 Repeatability

Repeatability estimates were based on 27 male lizards. All behavioural traits were repeatable (transitions: ICC = 0.43, *p* = .01; basking: ICC = 0.69, *p* < .001; perching: ICC = 0.56, *p* < .001). Among thermal preference variables, *T*_pref_CV (ICC = 0.56, *p* < .001) was repeatable. *T*_pref_ (ICC = 0.25, *p* = .096) and shuttling had low repeatabilities (ICC = 0.03, *p* = .43).

### 3.2 Thermal and behavioural traits

#### 3.2.1 Sample size

Thermal preference and behavioural traits were measured in 91 individuals: 62 males and 29 females. Of the 62 males, we sampled 37 territorial individuals and 25 non‐territorial males. Of the 29 females, we sampled 14 orange, 13 yellow, and 2 white (excluded from analysis due to low sample size) individuals.

#### 3.2.2 Trait variation between male status groups

**FIGURE 3:**
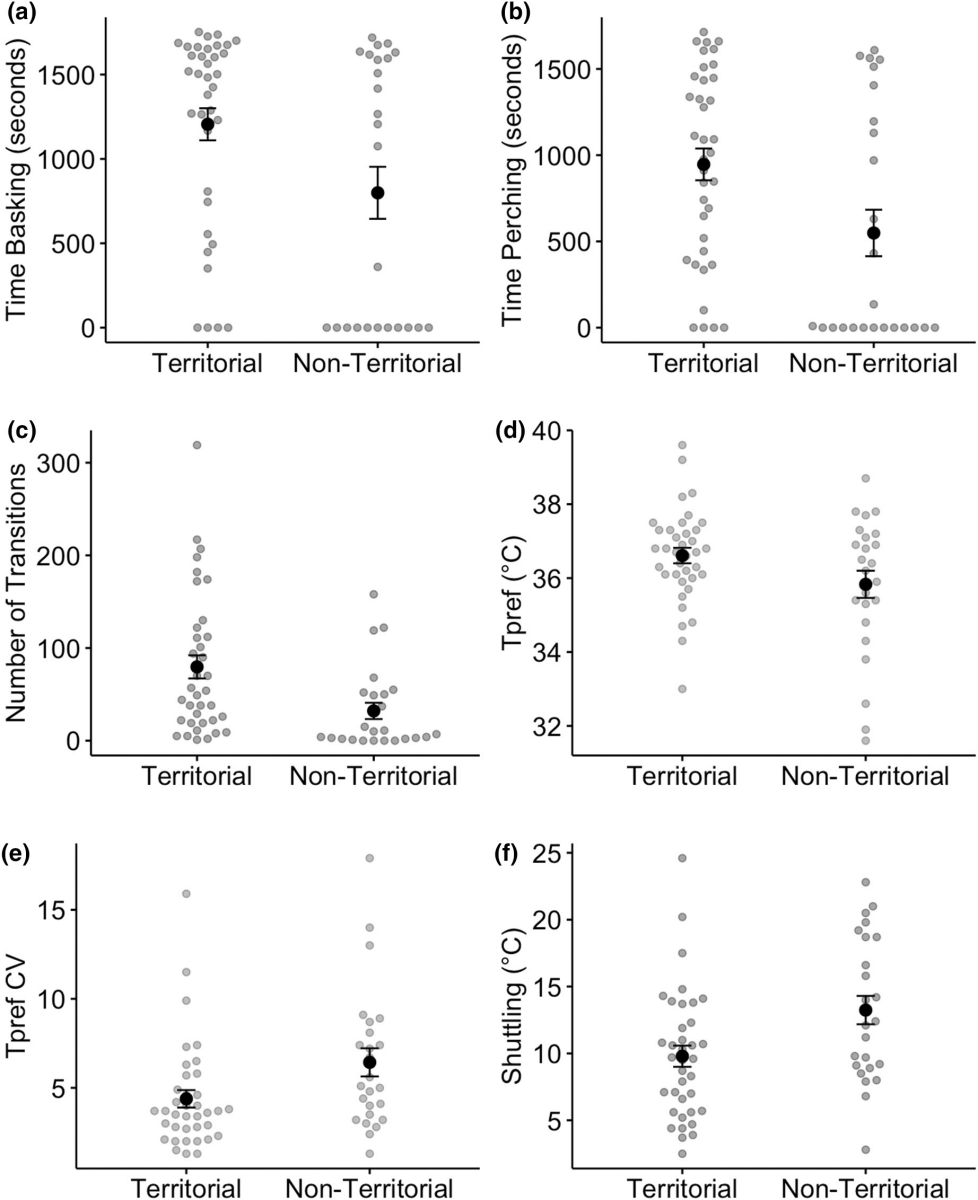
Variation in behavioural (a‐c) and thermal (d‐f) traits between territorial and non‐territorial males. All traits varied significantly between male status groups. Dark points and error bars represent mean and standard errors. Grey points represent individual values.

In males, behavioural and thermal trait expression differed between status groups (Pillai's Trace = 0.23, *F*_1,54_ = 2.7, *p* = .02). Territorial males were bolder and more exploratory than non‐territorial males. Territorial males also exhibited higher values for preferred body temperature than non‐territorial males and had lower *T*_pref_CV and shuttling values than non‐territorial males (Table 1; Figure 3). The effect size of all variables between male status groups was moderate (Table 1). Due to the significant differences between groups, all traits were considered for further analysis.

**TABLE 1:** Behavioural and thermal trait variation between territorial and non‐territorial male *Urosaurus ornatus.*

Variable	Male group	Mean ± SE	Test statistic	Degrees of freedom	*p* (FDR adjustment)	Cohen's *d*
Basking	Territorial	1205.2 ± 95.1 s	*H* = 4.1	1	.053	0.61
	Non‐Territorial	799.1 ± 154.3 s				
Perching	Territorial	946.6 ± 92.2 s	*H* = 5.9	1	.03	0.65
	Non‐Territorial	548.8 ± 134.9 s				
Exploration	Territorial	79.6 ± 12.5 transitions	*F* = 7.8	1,59	.03	0.73
	Non‐Territorial	32.1 ± 8.8 transitions				
*T*_pref_	Territorial	36.6 ± 0.21°C	*F* = 3.9	1,59	.053	0.51
	Non‐Territorial	35.8 ± 0.36°C				
*T*_pref_CV	Territorial	4.4 ± 0.49	*F* = 5.3	1,59	.03	0.60
	Non‐Territorial	6.4 ± 0.79				
Shuttling	Territorial	9.8 ± 0.79	*F* = 6.9	1,59	.03	0.69
	Non‐Territorial	13.2 ± 1.0				

*Note:* Territorial males were bolder and more exploratory, preferred higher body temperatures, selected a narrower range of body temperatures, and shuttled less in the thermal gradient compared to non‐territorial males.

#### 3.2.3 Trait variation between female morphs

**FIGURE 4:**
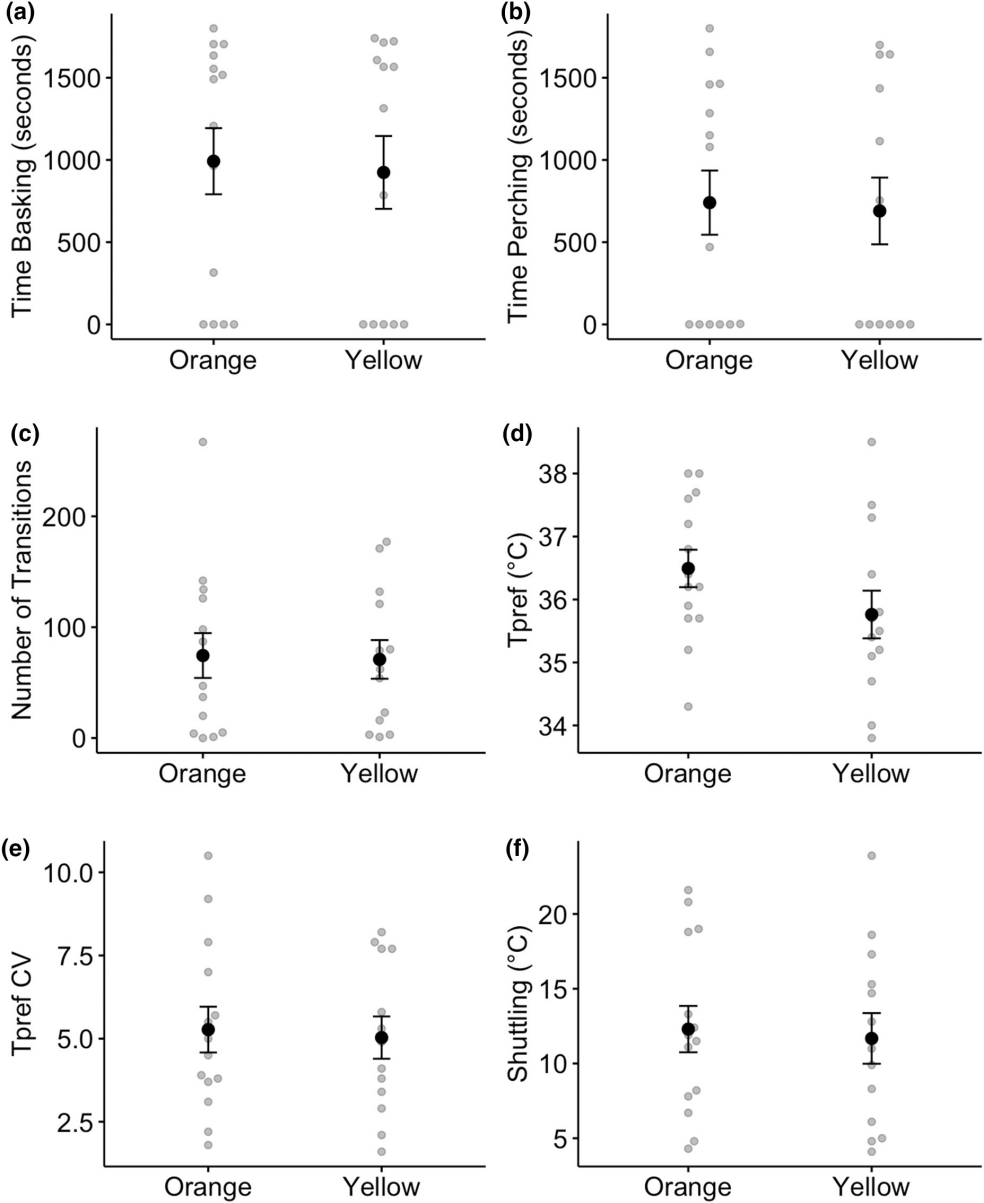
Variation in behavioural (a‐c) and thermal (d‐f) traits between orange and yellow females. No traits varied between female morphs. Dark points and error bars represent mean and standard errors. Grey points represent individual values.

**FIGURE 5:**
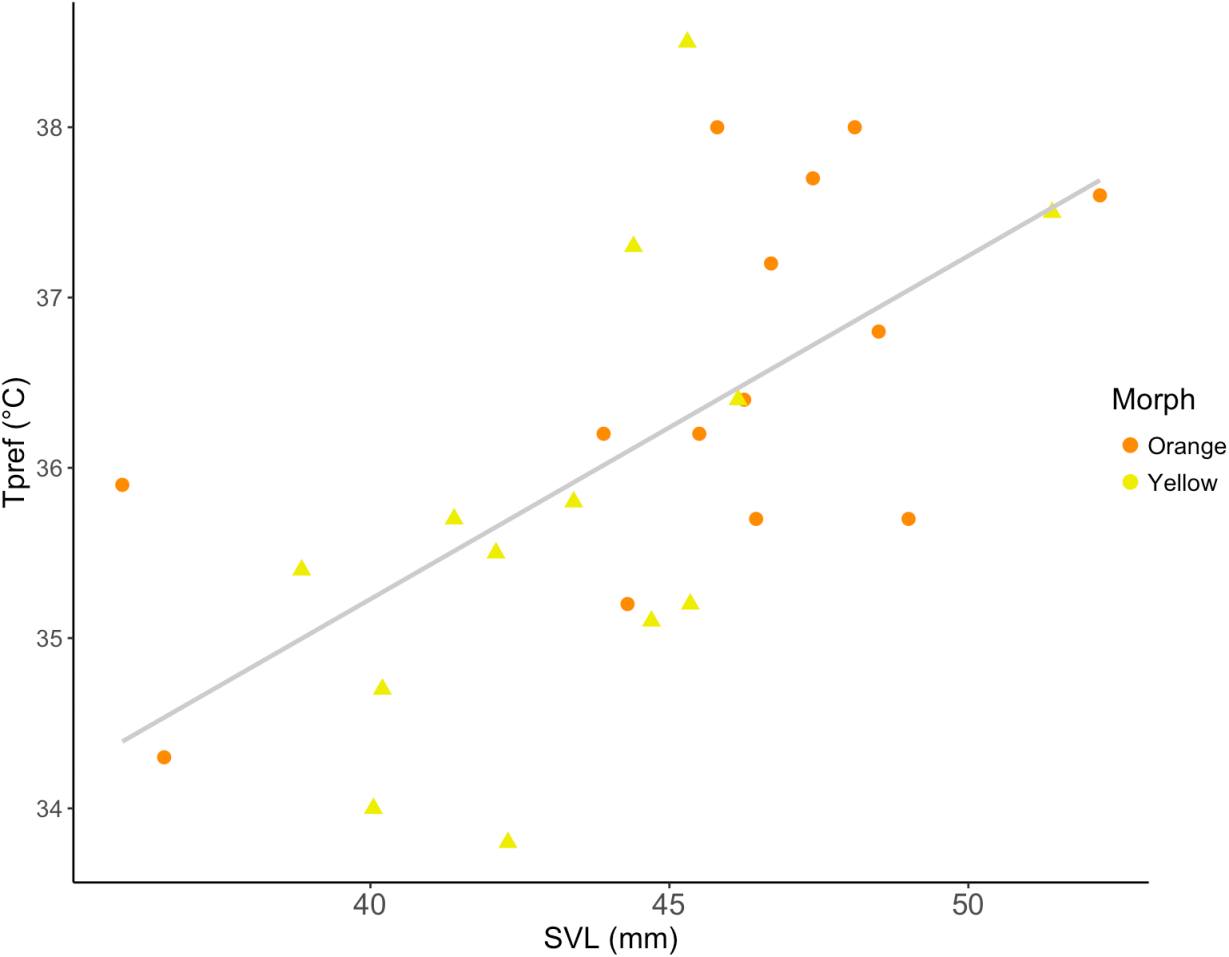
Larger female individuals prefer warmer body temperatures (*F*_1,25_ = 16.6, *p* < .001). There was no relationship between morph and SVL (*t*(25) = 1.2, *p* = .21).

In females, behavioural and thermal trait expression did not differ between orange and yellow morphs (Table 2; Figure 4). However, SVL influenced trait expression in females (Pillai's Trace = 0.47, *F*_1,19_ = 2.8, *p* = .04). Specifically, larger females preferred higher body temperatures (*F*_1,25_ = 16.6, *p* < .001, Figure 5). There was no significant difference in SVL between orange and yellow morphs (orange = 45.5 ± 1.2 mm, yellow = 43.5 ± 0.9 mm; *t*(25) = 1.2, *p* = .21).

**TABLE 2:** Behavioural and thermal trait variation between orange and yellow female *Urosaurus ornatus* morphs. No traits varied significantly between morph.

Variable	Female morph	Mean ± SE	Pillai's trace	*F*	Degrees of freedom	*p*
Basking	Orange	992.2 ± 75.3 s	0.06	0.22	1,19	.97
	Yellow	924.1 ± 79.9 s				
Perching	Orange	740.4 ± 73.1 s				
	Yellow	689.8 ± 73.2 s				
Exploration	Orange	74.4 ± 7.6 transitions				
	Yellow	70.9 ± 6.3 transitions				
*T*_pref_	Orange	36.5 ± 0.11°C				
	Yellow	35.8 ± 0.14°C				
*T*_pref_CV	Orange	5.3 ± 0.26				
	Yellow	5.0 ± 0.23				
Shuttling	Orange	12.3 ± 0.58°C				
	Yellow	11.7 ± 0.61°C				

### 3.3 PCA

**FIGURE 6:**
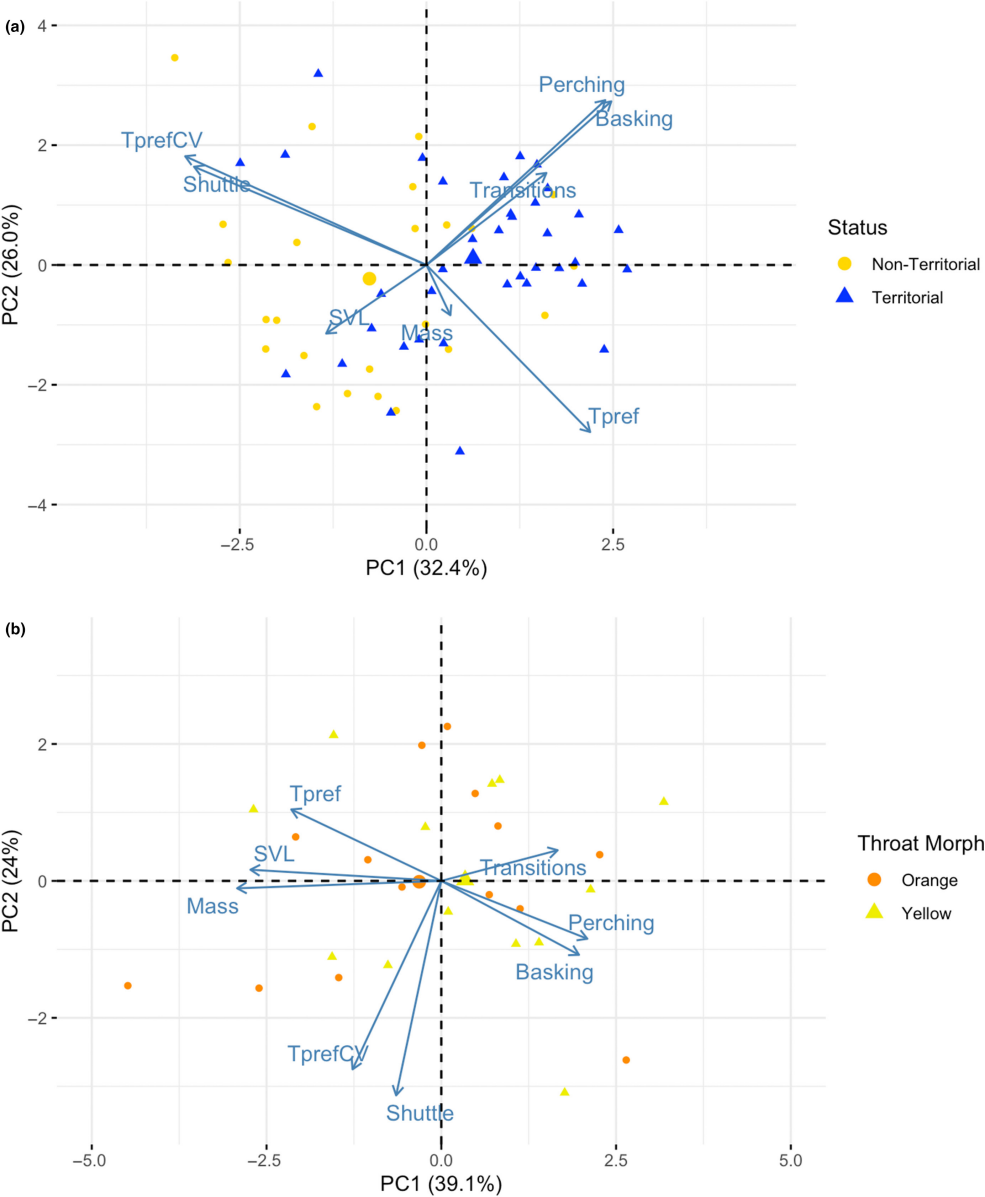
(a) Patterns of covariation among behavioural and thermal traits and body size based on the first two axes from a principal component analysis in males. Territorial and non‐territorial males differed in PC space, specifically on PC1 (*F*_1,60_ = 17.6, *p* < .001). The mean position of each status group is designated with a larger shape in the plot. (b) Patterns of covariation among behavioural and thermal traits and body size based on the first two axes from a principal component analysis in females. The mean position of each morph is designated with a larger shape in the plot. Orange and yellow females did not differ in PC space.

We assessed behavioural and thermal trait covariation in relation to male status and female morph using PCAs. In males (*N* = 62), we retained two PC axes based on the broken stick criterion (Jackson, 1993), which explained 58.4% of the total variation. The first PC axis explained 32.4% of the total variation and described covariation between thermal and behavioural traits, showing that males with high boldness and exploration scores had higher *T*_pref_ values and lower *T*_pref_CV and shuttling values. The second PC axis explained 26% of the total variance and showed that males with higher boldness scores had lower *T*_pref_ values (Table 3; Figure 6a). Territorial and non‐territorial males differed significantly in PC space (*F*_1,59_ = 8.9, *p* < .001), and specifically along PC1 (*F*_1,60_ = 17.2, *p* < .001).

**TABLE 3:** Loadings (variable coordinates/square root of eigenvalue) of behavioural and thermal preference traits and body size to PC Axes 1 and 2 in male lizards.

Variable	PC axis	
	1	2
Transitions	0.25	0.27
Basking	0.39	0.47
Perching	0.37	0.48
*T*_pref_	0.34	−0.48
*T*_pref_CV	−0.50	0.32
Shuttle	−0.49	0.29
SVL	−0.21	−0.20
Mass		−0.15
Eigenvalue	2.59	2.08
Percent variance explained	32.4%	26.0%
Cumulative variance explained	32.4%	58.4%

In females (*N* = 29), four PC axes were retained based on the broken‐stick criterion and accounted for 91.9% of the total variation. The first PC axis explained 39.1% of the total variation and had the greatest contributions from SVL and mass, where smaller females had lower *T*_pref_ values and were bolder. The second PC axis explained 24% of the total variation and was dominated by negative loadings from *T*_pref_CV and shuttling. The third PC axis explained 18% of the total variation and described larger females as bolder and preferring higher body temperatures. The fourth PC axis explained 10.8% of the total variation and was dominated by a positive loading from exploratory behaviour, where more exploratory females preferred higher body temperatures (Table 4; Figure 6b). There was no difference between female colour morphs in PC space (*F*_1,25_ = 0.04, *R* ^2^ = .002, *p* = .93).

**TABLE 4:** Loadings (variable coordinates/square root of eigenvalue) of behavioural and thermal preference traits and body size to PC Axes 1, 2, 3, and 4 in female lizards.

Variable	PC Axis			
	1	2	3	4
Transitions	0.29		0.14	0.87
Basking	0.34	−0.24	0.55	−0.15
Perching	0.36	−0.19	0.54	−0.14
*T*_pref_	−0.37	0.23	0.35	0.34
*T*_pref_CV	−0.22	−0.61	−0.16	0.29
Shuttle	−0.11	−0.69		
SVL	−0.47		0.38	
Mass	−0.50		0.30	
Eigenvalue	3.13	1.92	1.44	0.87
Percent variance explained	39.1%	24.0%	18.0%	10.1%
Cumulative variance explained	39.1%	64.1%	81.1%	91.9%

### 3.4 Tail loss

We completed boldness testing on 20 males with tail breaks and matched each male with his nearest neighbour without a tail break. In 15/20 nearest neighbour sets, the male with a tail break was significantly bolder (spent more time basking/perching after a simulated predation attack) than the male without a tail break (χ^2^ = 8.1, df = 1, *p* = .004). Among these 40 males, 25 were territorial and 15 were non‐territorial. While territorial males experienced higher rates of tail breaks (15/25) compared to non‐territorial males (5/15), the relationship between tail breaks and male status was not significant (χ^2^ = 1.7, df = 1, *p* = .19).

## 4 DISCUSSION

Colour polymorphic lizard species often exhibit behavioural variation between morphs that reflects alternative reproductive strategies and dominance hierarchies (Sinervo & Lively, 1996; Stuart‐Fox et al., 2021). Variation among morphs in other behaviours and in thermal traits is less studied. Here, we found that in *U. ornatus* males, behavioural and thermal trait expression covaried with morph‐associated territorial status. Territorial males were bolder and more exploratory than non‐territorial males. In addition, non‐territorial males exhibited greater amounts of shuttling behaviour and higher variation in preferred temperatures. In contrast, territorial males minimized movement while basking, resulting in higher preferred body temperatures and a lower CV. Male body size had no discernible influence on relationships between territorial status and trait expression. All behavioural traits and *T*_Pref_CV were repeatable in males. In females, colour morph had no discernible influence on behaviour or thermal traits and we found no significant covariation between any traits. However, larger females were selected for higher body temperatures in the thermal gradient.

Colour polymorphic species have been shown to occupy different environmental niches with varying thermal quality and access to resources such as food and mating opportunities (Calsbeek & Sinervo, 2002; Forsman & Åberg, 2008; Forsman et al., 2008; i de Lanuza & Carretero, 2018; Thompson et al., 2023). In *U. ornatus*, blue, yellow/blue, and orange/blue males are territory holders and dominant over yellow, orange, and orange/yellow morphs (Hews & Moore, 1995; Hover, 1985; Knapp et al., 2003; Moore & Thompson, 1990; Taylor & Lattanzio, 2016; Thompson & Moore, 1991b). Trophic and habitat niche partitioning also occurs between morphs. Territorial males are trophic specialists and utilize microhabitats that offer prey items at a higher trophic level and provide structural heterogeneity and high thermal quality. In contrast, non‐territorial males are trophic generalists and are often relegated to low‐quality habitat, especially when overall resources are plentiful (Lattanzio & Miles, 2014, 2016).

Our study provides insight into behavioural mechanisms that underly the ecological segregation and variation in microhabitat use between status groups. In other lizard species, bolder and more exploratory individuals occupy larger territories than shyer, less exploratory individuals. Bolder individuals also enjoy higher mating success and habituate to predators more quickly (Rodríguiz‐Prieto et al., 2011; Ward‐Fear et al., 2018). Increased territory size can enable dominant males to exploit higher‐quality habitat (Fox et al., 1981). Our finding that territorial male *U. ornatus* are bolder and more exploratory than non‐territorial males provides evidence that ecological niche segregation between colour morphs may be in part driven by variation in behavioural expression.

We also found that bolder males had significantly higher rates of tail loss than shyer males and that this relationship was not solely a consequence of territorial versus non‐territorial status. In addition to providing evidence that our laboratory‐based protocol is a reasonable representation of boldness in the field, this finding offers additional insight into ecological ramifications of boldness variation in this population of *U. ornatus*. Heightened boldness during activities including territory defence, courtship, and foraging carries increased predation risk. While tail autotomy can increase short‐term survivorship by avoiding mortality, several costs are associated with tail loss. Individuals that drop their tails lose access to high concentrations of lipids stored in the tail and incur energy costs growing them back, and tail loss can compromise dominance status, home range size, mating success, and immunity (Doughty et al., 2003; Kuo et al., 2013; Martin & Salvador, 1993; Salvador et al., 1995).

We documented significant thermal trait variation between territorial and non‐territorial male morphs. In addition to being bolder and more exploratory, territorial male morphs preferred higher body temperatures and maintained body temperatures in a narrower range than non‐territorial morphs. Despite the prevalence of colour polymorphisms in lizards, limited data exist pertaining to morph variation in preferred body temperature. We are aware of two other studies that report differences in thermal preference among morphs. Paranjpe et al. (2013) found that in *U. stansburiana*, yellow female morphs preferred higher body temperatures than orange and yellow/orange females. In addition, Thompson et al. (2023) demonstrated that orange *P. erhardii* morphs preferred lower temperatures than did white and yellow morphs, coinciding with variation in microhabitat use. The findings presented here are further evidence that variation in thermal preference should be considered in studies of polymorphic species. Importantly, variation in *T*_pref_ between morphs covaried with boldness and exploratory behavioural strategies consistent with the ecological niche separation between morph groups documented by Lattanzio and Miles (2016). We expected a heterogeneous environment to favour divergence in thermal preference coinciding with differences in microhabitat exploitation (Lelièvre et al., 2011). Indeed, behavioural differences observed between morphs could reflect the fact that they occupy microhabitats that vary in thermal quality. In addition to preferring higher body temperatures in the thermal gradient, territorial males selected for higher body temperatures in the field than non‐territorial males (average field *T*_b_ of territorial males: 34.1°C; average field *T*_b_ of non‐territorial males: 32.9°C). We suggest that the observed pattern of greater precision (lower *T*_pref_CV) associated with a higher *T*_pref_ and field active *T*_b_ may reflect territorial males excluding non‐territorial males from access to higher‐quality thermal environments, as maintenance of preferred body temperatures is easier for individuals in high‐quality habitat (Calsbeek & Sinervo, 2002; Waldschmidt & Tracy, 1983). In contrast, non‐territorial males demonstrated wider ranges of body temperatures while in the thermal gradient and used shuttling to a greater extent during thermoregulation. This divergence in thermoregulatory behaviour may be a consequence of observed ecological niche partitioning between morphs: non‐territorial males, which often occupy lower‐quality habitat than territorial males (Lattanzio & Miles, 2014, 2016), may have to rely on increased shuttling behaviour throughout a wider range of thermal conditions to achieve their preferred body temperatures.

Homogeneous patterns of trait variation in females suggest that colour polymorphism in female *U. ornatus* is not related to boldness, exploration, or exploitation of the thermal environment. Although some studies have documented behavioural and thermal trait variation among female lizard morphs (Paranjpe et al., 2013; Thompson et al., 2023), our findings are consistent with previous work indicating that in many cases, trait variation between sympatric female morphs coincides more with mate choice and reproductive traits than with behaviour. The few studies that have addressed variation between female morphs in *U. ornatus* demonstrate similar relationships. Lattanzio et al. (2014) found that yellow and orange females differ in mating preference, with orange females preferring dominant males and yellow females preferring subordinate males. Zucker and Boecklen (1990) described throat colour changes when females became gravid, suggesting that female throat colour was a good predictor of clutch size. Although our results indicate lack of behavioural variation between female morphs, future studies could investigate the influence of the interaction between female morph and environmental condition on *U. ornatus* reproductive behaviour; for example, in *U. stansburiana*, female morphs alter clutch size and egg mass differentially depending on the presence of same‐ and different‐morph neighbours (Svensson et al., 2001).

In any case, larger females preferred higher body temperatures than smaller females. Female *U. ornatus*, while less aggressive and territorial than males, establish and maintain territories (Mahrt, 1998), indicating that variation in thermal environments may be a cue for selecting microhabitats and establishing home ranges. It is unclear whether body size variation confers competitive advantage in microhabitat acquisition, as variation in female lizard thermal preference often does not correlate with body size (Beal et al., 2014; Cecchetto & Naretto, 2015). Instead, covariation between body size and *T*_pref_ in females may be associated with reproduction. We measured behavioural and thermal traits during the early part of vitellogenesis. Larger, gravid females may have preferred higher body temperatures to enhance the maturation of the eggs prior to oviposition (Webber et al., 2015).

Overall, our results demonstrate that alternative reproductive strategies in male *U. ornatus* coincide with behavioural and thermal trait covariation that may underly exploitation of variable thermal environments. Future studies could investigate behavioural and thermal trait variation in colour polymorphic species to bolster data on boldness, exploratory behaviour, and thermal preference variation among colour morphs given the ecological relevance of these traits (Brock & Madden, 2022; Carter et al., 2010; Chen et al., 2019; Rodríguiz‐Prieto et al., 2011; Thompson et al., 2023; Ward‐Fear et al., 2018). Elucidating trait variation in colour polymorphic species is critical for exploring how natural and sexual selection promote and maintain colour polymorphisms in natural populations (Chelini et al., 2021; Gray & McKinnon, 2007). Further, assessing distinct ecological roles among colour morphs and the trait variation that underlies niche segregation can inform how different morphs may be influenced by and respond to rapidly changing environments.

## AUTHOR CONTRIBUTIONS

**Tyler M. Goerge:** Conceptualization (equal); data curation (lead); formal analysis (lead); funding acquisition (lead); investigation (lead); methodology (equal); project administration (equal); resources (lead); software (lead); supervision (equal); validation (lead); visualization (lead); writing – original draft (lead); writing – review and editing (equal). **Donald B. Miles:** Conceptualization (equal); formal analysis (supporting); funding acquisition (supporting); methodology (equal); project administration (equal); resources (supporting); supervision (equal); writing – review and editing (equal).

## CONFLICT OF INTEREST STATEMENT

We declare no conflicts of interest.

## Data Availability

The data used for this study are available to download from Figshare at: https://doi.org/10.6084/m9.figshare.25979209. The “Morph Trait Variation” folder includes raw data (Trait_Variation_Morphs_Public_Data), an R script to repeat all analyses, all necessary .csv files derived from the raw data, and a metadata file.
